# Hippotherapy and neurofeedback training effect on the brain function and serum brain-derived neurotrophic factor level changes in children with attention-deficit or/and hyperactivity disorder

**DOI:** 10.20463/jenb.2017.0018

**Published:** 2017-09-30

**Authors:** Namju Lee, Sok Park, Jongkyu Kim

**Affiliations:** 1. Department of Sports Medicine, School of Sports Sciences, Jungwon University, Goesan-gun Republic of Korea; 2. Department of Sports Leadership, College of Natural Science, Kwangwoon University, Seoul Republic of Korea; 3. Aribio Sports Science Institute , Sungnam Republic of Korea

**Keywords:** Hippotherapy, Brain function, ADHD

## Abstract

**[Purpose]:**

The purpose of this study was to investigate the effect of hippotherapy and electroencephalography (EEG) neurofeedback on brain function and blood brain-derived neurotrophic factor (BDNF) level in children with attention-deficit or/and hyperactivity disorder (ADHD).

**[Methods]:**

Sixteen children with ADHD participated in this study and were randomly divided into 2 groups, a 1-time hippotherapy group (W1G, *n* = 8) and a 2-time hippotherapy group (W2G, *n* = 8). All the participants attended 8 weeks of hippotherapy program in the primary training, and then 7 children with ADHD attended 8 weeks of hippotherapy program combined with neurofeedback training in the secondary training. Blood BDNF levels were measured, and functional magnetic resonance imaging (fMRI) was performed. The EEG neurofeedback training program was used to train and measure psychological factors.

**[Results]:**

The combined effect of hippotherapy and neurofeedback on BDNF level showed a decreased tendency in W1G (pretraining, 1766.03 ± 362.54 pg/ml; posttraining, 1630.65 ± 276.70 pg/ml). However, the BDNF level of W2G showed an increased tendency (pretraining, 1968.28 ± 429.08 pg/ml; posttraining, 1976.28 ± 425.35 pg/ml). Moreover, combined training showed a significant group x repetition interaction in W1G (pretraining, 1436.57 ± 368.76 pg/ml; posttraining, 1525.23 ± 346.22 pg/ml; F = 3.870, *p* = 0.039). fMRI results showed that the left thalamus activity in both groups had a decreased tendency and a significantly lower change in W2G than in W1G (p < 0.05).

**[Conclusion]:**

This study confirmed a significant increase in blood BDNF level after combined training, which may induce brain function improvement in children with ADHD.

## INTRODUCTION

Attention-deficit or/and hyperactivity disorder (ADHD) has been known to usually evoke hyperactivity, impulsiveness, and aggression, and the incidence of ADHD higher in boys than in girls^1^. ADHD occurs in school-aged boys and girls, and is characterized by difficulty in social relationships and lower study achievement^2, 3^. In a previous report, Korean children showed a higher ADHD incidence (3–20%)^4^ than children in the United States (3–8%)^5^ and Japan (7–12%)^6^.

The physiological mechanism of ADHD shows that lower activity levels of dopamine D4 receptor gene and norepinephrine mainly induce functional reduction in the right and dorsal frontal lobe of the brain^7, 8^. The brain-derived neurotrophic factor (BDNF) plays a key role in nerve cell division of dopamine^9^. Moreover, dopamine is involved in the regulation of the posterior attention system (PAS) related to action, memory, judgment, plan, and response. Meanwhile, norepinephrine is involved in the regulation of the anterior attention system (AAS) related to stimulus selection and choice, and attention distribution, maintenance, and change. Accordingly, malfunction of PAS and AAS may cause severe problems in execution functions such as personal relationships and coordination^10, 11^. Malfunction in PAS and AAS activates slow brainwaves via quantitative electroencephalography (QEEG). Therefore, activating the theta-to-beta wave ratio would be useful to improve physiological symptoms in children with ADHD^12, 13, 14^. In addition, children with ADHD need to have medical treatment and management as early as possible because only 20% of these children recover as they age and most continue to have ADHD through their adulthood^8^.

Methylphenidate and amphetamine are drugs used for the treatment of ADHD; however, approximately 20% of these medicines have been reported to evoke side effects such as hypertension, sleep disorder, and mood disorder, and just temporarily reduce ADHD symptoms^15, 16^. Thus, exercise therapy for children with ADHD has been suggested to activate brain function^17^. A previous study reported that exercise therapy and/or physical activity combined with cognitive function training for children with ADHD positively affected ADHD^18^. Horseback riding or hippotherapy has been a useful therapy for enhancing neuromuscular function since the 1970s in Europe and the United States^19^. Use of animals such as horses in therapy would be effective for positively changing psychological factors, social positioning act, quality of life, and movement function in children with ADHD^20, 21^. Horseback riding has been known to be effective as a high-intensity physical activity (>4 METs)^22^. Thus, hippotherapy would apply to functionally improving behavioral modification and psychological factors in children with ADHD. In addition, previous studies confirmed that above-moderate-intensity hippotherapy was positively effective for functional improvement in children with ADHD^17, 23^.

EEG neurofeedback training has been defined as a comprehensive training system by changing the brain function of children with ADHD^24^ and can generally be applied as a treatment method for enhancing brain function in children with ADHD because it is programmed for brain function improvement^12, 25^. Recent Internet technology uses a server-based long-distance learning system that enables patients to easily use the EEG neurofeedback training program and maximizes patients’ participation at home. In addition, this would be practically applicable for better therapeutic results in children with ADHD. Therefore, the purpose of this study was to investigate the combined effect of hippotherapy and EEG neurofeedback on brain function and blood BDNF level in children with ADHD.

## METHODS

### Participants

The study participants were patients with ADHD who were recruited from the pediatric psychiatry departments of G university and C university hospitals. Parents, physicians, and children with ADHD understood the study purpose and signed the study consent form. Previous medical history information was collected, and a psychological medical examination was conducted for calculating the attention quotient (AQ) for selecting the study patients. To examine the changes in serum BDNF level and brain function in children with ADHD followed by the primary training, 16 participants were divided into the “one time per week” group (W1G, n = 8) and the “two times per week” group (W2G, n = 8) during the 8-week training period. The secondary training was designed for W1G and combined with neurofeedback training, with 7 children with ADHD as participants (Table 1).

**Table 1.** AGE accumulation in tissues during aging.

Group	Age, years	Height, cm	Weight, kg
W1G	11.75 ± 1.28	143.56 ± 12.84	35.69 ± 10.75
W2G	12.00 ± 1.51	147.41 ± 15.77	40.40 ± 11.84
Combined training group	11.42 ± 0.98	144.71 ± 14.46	31.49 ± 13.47

The values are presented as mean ± SD.

W1G: “1 time per a week participation” group; W2G: “2 times per a week participation” group; combined training group: W1G + neurofeedback training.

### Hippotherapy program

#### Primary training

**Figure 1.**
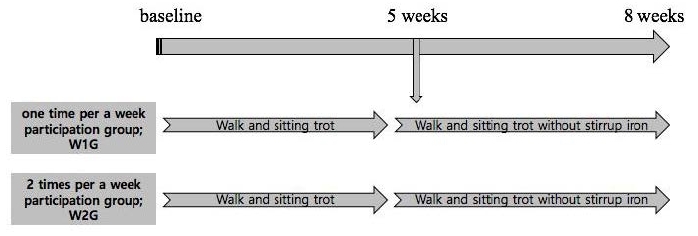
Primary training

Primary training was conducted to investigate the changes in serum BDNF level and brain function in the children with ADHD, followed by the 1- and 2-time hippotherapy programs during the 8-week training period. The participants in W1G and W2G performed the walk and sitting trot training during the first 4 weeks and the walk and sitting trot training without a stirrup iron after 5 weeks of training (Figure 1).

#### Secondary training

**Figure 2.**
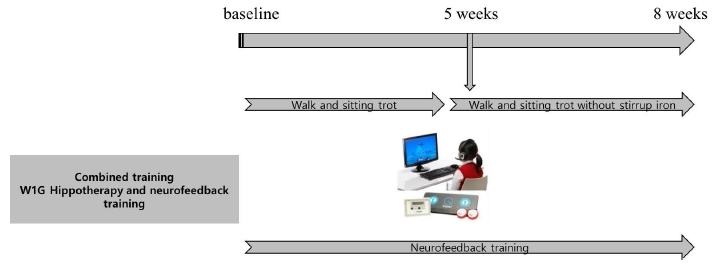
Combined secondary training

In W1G, training was combined with neurofeedback training. To examine the changes in serum BDNF level and brain function, the prefrontal lobe of the brain was trained using the NH neurofeedback system through the sequential bipolar montage for 30 minutes. The brain wave range was set at 45 Hz (Figure 2).

### Blood BDNF and functional magnetic resonance imaging analysis

#### Blood BDNF analysis

Blood BDNF level was measured at baseline and after the hippotherapy and combination trainings. Blood BDNF level was analyzed using human enzyme-linked immunosorbent assay kits (AB Frontier). Blood was collected from the forearm venous vein, and the collected blood was stored in serum tubes. It was centrifuged at 1000×g (relative centrifuged force) for 15 minutes, and 0.1 ㎖ of separated serum was analyzed using the Microplate model 680 (Bio-Rad, USA). Then, the blood sample was analyzed using a human BDNF kit, followed by the ELISA method. The BDNF level was calculated by measuring optical density.

#### Brain function test using functional magnetic resonance imaging

To measure brain function, functional magnetic resonance imaging (fMRI) was used. A 3.0-Tesla GE MR scanner (General Electric Medical Systems, Milwaukee, WI) was used to check the anatomical structure of the brain. T-1 imaging as the 3-D spoiled gradient echo pulse sequence (SPGR) was also used to scan 240 sagittal images of the brain. A T-2 image scan was used to check the brain structure. A fMRI scan was conducted using the quadrature-type head coil, and MR sequence was shown (Table 2).

**Table 2.** Magnetic resonance (MR) sequence.

Technique	MR Sequence
Functional images	GRE-EPI (TR/TE/flip angle, 3000 ms/30 ms/90; FOV, 200 mm; matrix, 64 × 64; slice, 20; slice thickness, 5 mm; total slices, 40; coronal view
High-resolution anatomical images	3-D SPGR (TR/TE/flip angle, 22 ms/4 ms/40; FOV, 240 mm; slice, 120; slice thickness, 1.5 mm; matrix, 256 × 256; scan time, 9 min)

GRE-EPI, gradient-echo echo-planar imaging; TR: repetition time; TE: echo time; FOV: field of view; 3-D SPGR: three-dimensional (3-D) spoiled gradient-recalled echo.

### EEG and comprehensive attention test followed by the combined training

#### EEG (electroencephalogram) test

EEG was used to test brain waves and Neuro H. Neurofeedback System was used. Neuro H. System is a portable 2 Channel System digital brain wave measuring instrument and it measures the both frontal robes by using the Sequential Bipolar Montage. Brain Quotient (BQ) program was used to test brain waves.

#### Comprehensive attention test measurement

Comprehensive attention test (CAT) is a standardized attention test that includes 6 subcategories, namely visual simple selection attention capacity, auditory simple selection attention capacity, suppression continuous attention capacity, interference selection attention capacity, division attention capacity, and job memory capacity. CAT is a computer-based program that takes 30–40 minutes to complete and calculates the tester’s score. CAT can report emotion and behavioral scores of children with ADHD. This measurement was used only in the secondary training.

### Data Analysis

Descriptive statistics were calculated using the means and the standard deviations from the collected data. The two-way mixed analysis of variance (group × repetition) was used to test the changes in serum BDNF level after the primary training. In addition, to examine the fMRI results for brain function changes, a paired-sample T test was conducted by converting image data into numeric data and to compare the difference between the baseline and after the 8-week training. To confirm the efficacy of the secondary training, a paired-sample T test was conducted. The significance level in all the tests was set at p < 0.05.

## RESULTS

### Primary training

#### BDNF level

**Figure 3.**
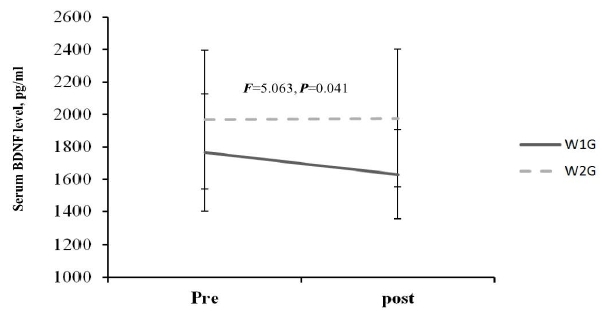
Change in serum BDNF level after primary training

**Figure 4.**
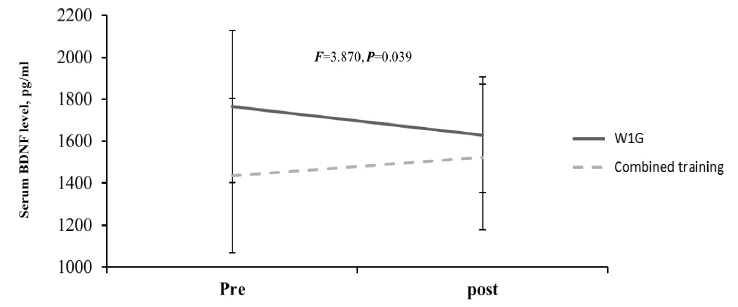
Change in serum BDNF level after combined secondary training

The blood BDNF level change after hippotherapy based on hippotherapy frequency showed a decreased tendency in W1G from before (1766.03 ± 362.54 pg/ml) to after training (1630.65 ± 276.70 pg/ml). By contrast, the blood BDNF level in W2G showed an increased tendency from before (1968.28 ± 429.08 pg/ml) to after training (1976.28 ± 425.35 pg/ml), which showed a significant interaction (F = 5.063, p = 0.041; Figure 3.). Considering the mean difference between the groups at baseline, delta differences (pre-post) were compared, and delta was increased in W2G but was decreased in W1G, with a significant difference between the groups (p < 0.05). Moreover, W1G combined with neurofeedback training showed an increased blood BDNF level from before (1436.57 ± 368.76 pg/ml) to after (1525.23 ± 346.22 pg/ml), which showed a significant group × repetition interaction (F = 3.870, p = 0.039; Figure 4).

#### Functional magnetic resonance imaging

Brain activity changes after hippotherapy showed a decreased tendency in the right precentral cortex in W1G and W2G; otherwise, no significant difference was observed. On the contrary, the left thalamus activity in both groups showed a decreased tendency and a significant lower change in W2G than in W1G (p < 0.05; Table 3).

**Table 3.** Brain function changes based on hippotherapy frequency

Activation area	Group	Pretraining	Posttraining	F (p)
Right precentral cortex	W1G	0.388 ± 0.117	0.379 ± 0.130	0.417 (0.529)
	W2G	0.408 ± 0.115	0.407 ± 0.106	
Left thalamus	W1G	0.464 ± 0.162	0.451 ± 0.174	11.79 (0.004)
	W2G	0.493 ± 0.099	0.377 ± 0.095	

All data are shown as mean ± SD.

W1G: “1 time per week” participation group; W2G: “2 time per week” participation group.

### Secondary training

#### Combined effect of hippotherapy and neurofeedback training on BDNF level

As shown in Figure 4, the combined effect of hippotherapy and neurofeedback on BDNF level showed a decreased tendency in W1G (pretraining, 1766.03 ± 362.54 pg/ml; posttraining, 1630.65 ± 276.70 pg/ml). By contrast, the BDNF level in W2G showed an increased tendency (pretraining, 1968.28 ± 429.08 pg/ml; posttraining, 1976.28 ± 425.35 pg/ml). Moreover, combined training showed a significant group × repetition interaction in W1G (pretraining, 1436.57 ± 368.76 pg/ml; posttraining, 1525.23 ± 346.22 pg/ml; F=3.870, p = 0.039).

#### Combined effect of hippotherapy and neurofeedback training on brain function

Brain wave (BQ) changes after the 8-week combined training with hippotherapy and neurofeedback did not induce significant changes in basic rhythm (Left·Right), self-control index, attention index (Left·Right), activation index (Left·Right), emotion index, anti-stress index (Left·Right), left and right brain balance, and brain index. This might have resulted from taking medication during the 8-week training, and emotional expressions seem to be absent at the pretest (Table 4).

**Table 4.** Combined training effect on brain wave changes (BQ)

Variables	Pretraining	Posttraining	t	p
Basic rhythm (left)	74.23 ± 6.84	75.07 ± 9.04	−0.189	0.856
Basic rhythm (right)	75.40 ± 9.70	72.85 ± 10.20	0.363	0.729
Self-control index	48.47 ± 14.28	51.93 ± 13.50	−0.393	0.708
Attention index (left)	66.67 ± 9.54	64.07 ± 8.35	0.449	0.669
Attention index (right)	64.18 ± 12.60	65.97 ± 9.23	−0.347	0.741
Activation index (left)	76.11 ± 9.56	71.82 ± 14.88	0.509	0.629
Activation index (right)	77.44 ± 13.87	74.06 ± 12.39	0.374	0.721
Emotion index	83.82 ± 2.45	82.68 ± 3.45	0.883	0.411
Anti-stress index (left)	75.27 ± 9.28	75.36 ± 17.38	−0.012	0.991
Anti-stress index (right)	74.44 ± 9.25	74.71 ± 15.86	−0.045	0.965
Left and right brain balance index	77.04 ± 9.48	78.54 ± 11.14	−0.348	0.740
Brain index	72.10 ± 5.12	71.55 ± 5.67	0.162	0.877

All data are shown as mean ± SD.

W1G: “1 time per week” participation group; W2G: “2 time per week” participation group.

As shown in Table 5, the left and right bilateral middle frontal cortex area and left precentral cortex showed an increased brain function activity after combined training.

**Table 5.** Brain function activation area after combined training

Region	p Value	t	Coordinate
Left bilateral middle frontal cortex	0.001	3.09	(−42, 36, 15)
Right bilateral middle frontal cortex	0.001	5.79	(48, 42, 15)
Left precentral cortex	0.001	5.59	(−51, 3, 36)

#### Combined effect of hippotherapy and neurofeedback training on CAT score

As shown in Table 6, the combined effect of hippotherapy and neurofeedback training showed a significant decrease in false-alarm error of suppression continuous attention capacity test (t = 2.66, p = 0.04). An improved tendency of false-alarm error in the visual (t = 1.58, p = 0.17; t = 0.32, p = 0.76) and auditory simple selection attention capacity tests (t = 0.48, p = 0.65; t = 1.07, p = 0.33; t = 0.35, p = 0.74) were observed. However, a significant decrease in reaction time in the auditory simple selection attention capacity test (t = −2.66, p = 0.04) was found.

**Table 6.** Comprehensive attention test changes after combined training

Variables		Pretraining	Posttraining	t	p
Visual simple selection attention capacity	Omission error	93.86 ± 10.73	101.14 ± 3.48	−1.79	0.12
	False-alarm error	101.29 ± 6.05	98.00 ± 5.74	1.58	0.17
	Reaction time (RT)	76.14 ± 16.05	91.57 ± 8.38	−2.06	0.09
	RT standard deviation	63.57 ± 23.12	82.71 ± 14.14	−1.84	0.12
Auditory simple selection attention capacity	Omission error	99.29 ± 4.35	98.00 ± 4.58	0.48	0.65
	False-alarm error	101.43 ± 4.39	94.14 ± 17.52	1.07	0.33
	Reaction time (RT)	87.00 ± 8.16	94.29 ± 10.09	−2.66	0.04
	RT standard deviation	86.29 ± 17.40	90.14 ± 21.71	−0.72	0.50
Suppression continuous attention capacity	Omission error	94.14 ± 7.03	95.14 ± 5.27	−0.36	0.73
	False-alarm error	99.57 ± 11.94	93.00 ± 13.00	2.66	0.04
	Reaction time (RT)	90.29 ± 16.53	91.29 ± 11.18	−0.20	0.85
	RT standard deviation	91.43 ± 12.91	88.86 ± 9.06	0.76	0.47
Interference selection attention capacity	Omission error	76.86 ± 20.99	88.57 ± 17.63	−1.49	0.19
	False-alarm error	97.29 ± 14.93	98.14 ± 8.23	−0.14	0.89
	Reaction time (RT)	82.57 ± 12.50	87.43 ± 9.57	−1.22	0.27
	RT standard deviation	85.43 ± 11.27	88.29 ± 11.38	−1.06	0.33

All scores are shown in attention quotient.

**Figure 5.**
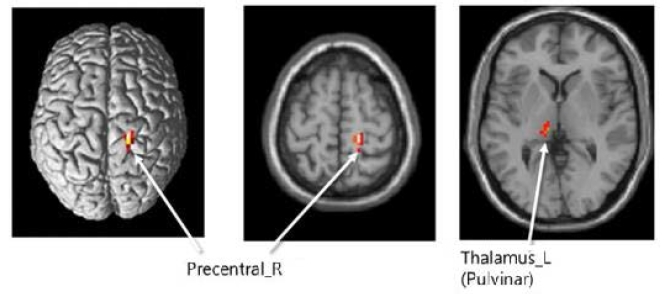
Brain function image produced using fMRI image recording

## DISCUSSION

This study suggests that hippotherapy might induce neurotransmitter activation in children with ADHD because they usually have lower neurotransmitter activation^7, 8^ and are expected to have increased activities in the right and dorsal frontal lobes. This study proved that the left thalamus activity in W2G was significantly higher than that in W1G. However, participation twice per week in the hippotherapy program may practically cause a cost-effectiveness problem; thus, children with ADHD are recommended to participate once per week in the hippotherapy program combined with neurofeedback training because neurofeedback training has a comparably lower cost and is as fun and interesting as a game for children. In this study, we found a significant job memory increase of the left bilateral middle frontal cortex, right bilateral middle frontal cortex, and left precentral cortex after by hippotherapy and neurofeedback training. Moreover, this study confirmed that the decision-making area of the brain activity was increased during task performance.

This study also suggests that skeletal muscle improvement in children with ADHD throughout horseback riding might regulate histone deacetylase (HDAC) from skeletal muscles. McGee et al.^26^ also suggested that regular physical activity might induce HDAC regulation and thus blood BDNF activation. These study results showed that the blood BDNF level in W1G combined with neurofeedback training was significantly increased in comparison with that in W2G. This study result may have been induced by the HDAC regulation caused by the muscle contraction after hippotherapy participation and brain function changes at the cell level after neurofeedback training^12, 24, 25^.

Increased left thalamus activity in W2G may be effective in suppressing caloric intake and might be expected to be effective for reducing oversupplied caloric intake in children with ADHD. This study did not investigate the hyperactivity pattern in children with ADHD and may not clearly present behavioral pattern changes via the hippotherapy program combined with neurofeedback training. Nevertheless, the blood BDNF changes after combined training showed a possibility to contribute to behavioral pattern changes throughout the hippotherapy program combined with neurofeedback training. Mattson (2010)^27^ insisted on a negative association between dietary energy intake and blood BDNF level, which supports our finding. Our study results showed a significant increase in blood BDNF level after combined training, which may induce brain function improvement in children with ADHD. No previous study related to the present study has been conducted; thus, we cannot clearly compare our study results to previous study results. However, we can expect a positive combined effect of hippotherapy and neurofeedback training because this study found significant cerebral cortex activity changes. Moreover, the present study results showed no significant difference in serum BDNF level between the primary and secondary trainings. However, this would be considered as a crucial factor for understanding the overall study results. We can assume that the sensitivity of serum BDNF concentration was affected by the analytical method by removing plasma protein such as fibrinogen, which resulted in a decreased BDNF concentration and was affected by the muscle contraction in children with ADHD before blood drawing. In relation with this assumption, Cho et al.^28^ reported that muscle contraction could induce platelet activation and affect changes in serum BDNF level changes. To clarify this assumption, an adjusted method for examining serum BDNF level changes, followed by the physical activity level, in children with ADHD should be considered in future intervention studies.

This study has two limitations. First, the ADHD level of each participant might be inconsistent, which might have negatively affected the study results. Second, the progress of each participant in hippotherapy differed because of the different levels of learning ability. This might have caused a variation in exercise intensity, although the study set the same exercise intensity. We recommend that future studies consider consistency of ADHD level and unified exercise intensity application for better understanding of the effects of hippotherapy and neurofeedback training on children with ADHD.

## CONCLUSION

Enhanced brain function was observed in the children with ADHD as their participation frequency in hippotherapy increased. However, a cost-effectiveness problem may be evoked as hippotherapy participation frequency is increased. Therefore, participation once per week in the hippotherapy program combined with neurofeedback training would optimize cost-effectiveness and brain function enhancement in comparison with participation twice per week in the hippotherapy program. This study suggests that hippotherapy combined with various psychological interventions would be useful for improving brain function in children with ADHD.
